# Application of an iPSC‐Derived Organoid Model for Localized Scleroderma Therapy

**DOI:** 10.1002/advs.202106075

**Published:** 2022-03-22

**Authors:** Jie Ma, Wei Li, Ruiyuan Cao, Dunqin Gao, Qiyu Zhang, Xiao Li, Biyou Li, Luye Lv, Mansheng Li, Junyi Jiang, Yujie Wang, Jun Li, Zhihong Wu, Yunping Zhu, Wu Zhong, Shuyang Zhang, Ling Leng

**Affiliations:** ^1^ State Key Laboratory of Proteomics Beijing Proteome Research Center National Center for Protein Sciences (Beijing) Beijing Institute of Lifeomics Beijing 102206 China; ^2^ National Engineering Research Center for the Emergency Drug Beijing Institute of Pharmacology and Toxicology Beijing 100850 China; ^3^ Stem Cell and Regenerative Medicine Lab Department of Medical Science Research Center State Key Laboratory of Complex Severe and Rare Diseases Translational Medicine Center Peking Union Medical College Hospital Chinese Academy of Medical Sciences and Peking Union Medical College Beijing 100730 China; ^4^ Department of Dermatology and Venereology Peking Union Medical College Hospital Chinese Academy of Medical Sciences and Peking Union Medical College Beijing 100730 China; ^5^ Basic Medical School Anhui Medical University Anhui 230032 China; ^6^ Institute of NBC Defense Beijing 102205 China; ^7^ Department of Cardiology Peking Union Medical College Hospital Chinese Academy of Medical Sciences and Peking Union Medical College Beijing 100730 China

**Keywords:** epithelial and mesenchymal organoid, human induced pluripotent stem cells, localized scleroderma, proteomics, skin

## Abstract

Localized scleroderma (LoS) is a rare chronic disease with extensive tissue fibrosis, inflammatory infiltration, microvascular alterations, and epidermal appendage lesions. However, a deeper understanding of the pathogenesis and treatment strategies of LoS is currently limited. In the present work, a proteome map of LoS skin is established, and the pathological features of LoS skin are characterized. Most importantly, a human‐induced pluripotent stem cell‐derived epithelial and mesenchymal (EM) organoids model in a 3D culture system for LoS therapy is established. According to the findings, the application of EM organoids on scleroderma skin can significantly reduce the degree of skin fibrosis. In particular, EM organoids enhance the activity of epidermal stem cells in the LoS skin and promotes the regeneration of sweat glands and blood vessels. These results highlight the potential application of organoids for promoting the recovery of scleroderma associated phenotypes and skin‐associated functions. Furthermore, it can provide a new therapeutic alternative for patients suffering from disfigurement and skin function defects caused by LoS.

## Graphical abstract

A proteome map of localized scleroderma (LoS) skin is established. A human‐induced pluripotent stem cell‐derived epithelial and mesenchymal organoids model is constructed and applied for LoS therapy. The study results highlight the potential application of organoids for promoting the recovery of scleroderma associated phenotypes and skin‐associated functions.

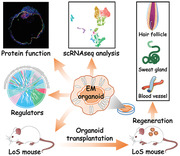

## 1 Introduction

Localized scleroderma (LoS) is a rare chronic connective tissue disease, confined to the skin and/or underlying tissues.^[^ ^1^ ^]^ They are characterized by skin thickening, plaque morphea, and hard, shiny skin involving the superficial dermis down to the fascia and muscle.^[^ ^2^, ^3^ ^]^ It has three pathological features: vasculopathy, immune system abnormalities, and fibrosis.^[^ ^4^, ^5^ ^]^ Among them, atrophic sweat glands, bullae, and necrotic keratinocytes at the dermoepidermal junction can be observed in skin lesions.^[^ ^4^ ^]^ However, these conditions are difficult to diagnose and treat effectively. LoS is considered an inflammatory fibrotic disease. Serology and histopathology of LoS reveal the interplay between circulating cytokines, immune cell infiltrates, and increased extracellular matrix (ECM) and sclerosis.^[^ ^6^, ^7^ ^]^ With the innovation of stem cell therapy, mesenchymal stromal/stem cells (MSCs) such as adipose‐derived stromal/stem cells and bone marrow‐derived MSCs have been utilized to modulate immune function and fibrosis of LoS.^[^ ^8^, ^9^ ^]^ In tissues with a regenerative capacity for wound repair, the skin needs to mobilize various cell types from the epidermis and dermis to promote its self‐renewal and regeneration during skin homeostasis and injury. Thus, it is vital to establish an ideal model composed of a variety of cells in an in vitro culture system, to find a potential therapeutic method for LoS. Here, we used human‐induced pluripotent stem cells (hiPSCs) to differentiate into an epithelial and mesenchymal (EM) organoids model in a 3D culture model for LoS treatment, which could provide potential guideline on the application of stem cells treatment for LoS.

## 2 Results

### 2.1 Pathological Characteristics of Skin Involved in Scleroderma Mice

**Figure 1:**
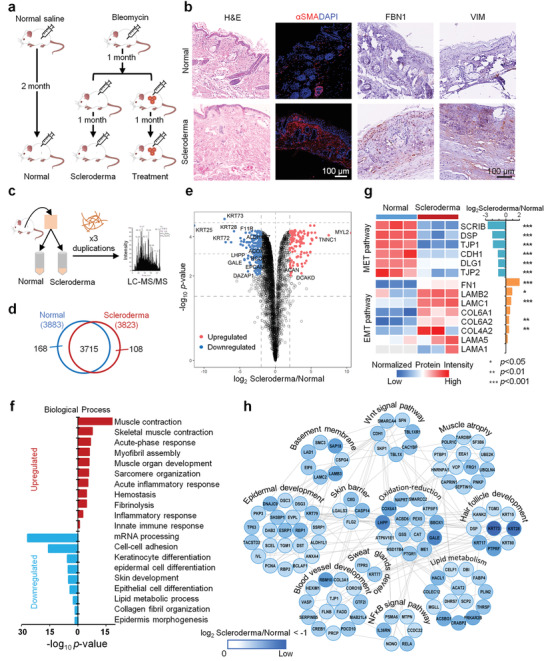
Quantitative proteome profiling of protein signatures in scleroderma mouse skin. a) Overview of study designs of LoS mouse model construction and organoid treatment. b) Hematoxylin and eosin (H&E) and immunochemical stainings of scleroderma and normal skins (scale bar: 100 µm). c) Schematics of the proteomics analysis used to evaluate scleroderma (*n* = 3) and normal (*n* = 3) mouse skin tissues. d) Overlap of the proteins identified in normal and scleroderma samples. e) Volcano plot of the −log_10_ *p*‐value versus the log_2_ protein abundance comparisons between scleroderma and normal mouse skin tissues. Proteins outside the significance threshold lines (−log_10_ *p‐*value > 2 and log_2_ Scleroderma/Normal > 1 or < −1) are shown in red (upregulated) and blue (downregulated). f) Biological process analysis of the upregulated and downregulated proteins in the skin of scleroderma mice versus normal mice. g) Heatmap represents the MET and EMT characterization of scleroderma (*n* = 3) and normal (*n* = 3) groups. Red and blue boxes indicate the normalized intensity of the enriched and depleted proteins, respectively. The histogram represents the log2 ratio of protein intensities between the scleroderma and normal groups. Asterisks indicate statistical significance, determined based on the Benjamini–Hochberg (BH)‐adjusted *p*‐value from pairwise comparison with a moderated t‐test (* *p* < 0.05; ** *p* < 0.01; *** *p* < 0.001). h) Interaction network of downregulated proteins in the skin of scleroderma mice versus normal mice. Functional clusters revealing the skin‐associated functions that are dysregulated in the scleroderma group compared to the normal group.

A localized scleroderma (LoS) mouse model was established via bleomycin injections for one month to simulate its pathological features (**Figure** **1**a). From a macroscopic point of view, the skin color was lighter and local lesions were sclerotic and atrophic, with a center slightly concave (Figure S1a, Supporting Information). Histopathology showed extensive fibrosis in the dermis and dense inflammatory infiltrates (CD4+ cells, CD8+T cells, and F480+macrophages) in the collagen bundles of the dermis (Figure 1b; Figure S1b, Supporting Information). Besides, *α*‐smooth muscle actin (*α*SMA), Fibulin 1 (FBN1), and vimentin (VIM) which are often used to identify pathologic fibroblasts and myofibroblasts were highly expressed in the dermis of scleroderma skin, compared to the control group (Figure 1b). However, other important organs and the skin without bleomycin injection were not involved (Figure S1c, Supporting Information). Next, a quantitative proteomics approach was used to analyze the pathological features of scleroderma skin tissues (Figure 1c and Table S1, Supporting Information). The results showed that the protein intensities varied by seven orders of magnitude (Figure S2a, Supporting Information). There were good correlations between the duplicated datasets within each group (Figure S2b, Supporting Information). A total of 3991 proteins were identified, including 3883 in normal skin and 3823 in scleroderma skin (Figure 1d). Among them, 3715 proteins were identified in both datasets, with 947 proteins differentially expressed between scleroderma mice and normal controls (Figure 1e and Table S2, Supporting Information).

Biological process analysis showed the upregulated proteins in mice with scleroderma were highly enriched in muscle contraction and development, myofibril assembly, and wound healing, including hemostasis, fibrinolysis, inflammatory, and immune responses, indicating that the skin of scleroderma mice exhibited myofibrosis and inflammatory infiltration (Figure 1f). The core ECM proteins, including 9 collagens, 6 proteoglycans, and 39 glycoproteins were upregulated in scleroderma mouse skin, according to the Matrisome database (Figure S3a and Table S3, Supporting Information).^[^ ^10^ ^]^ Among them, epithelial–mesenchymal transition (EMT) associated proteins including laminins, fibronectin 1, and type IV/VI collagens were also found upregulated (Figure 1g). However, mesenchymal–epithelial transition (MET) associated proteins such as SCRIB, DSP, TJP1, CDH1, DLG1, and TJP2 were downregulated in scleroderma skin, indicating the high severity of skin fibrosis in mice with scleroderma. Furthermore, the downregulated proteins were highly enriched in cell adhesion, lipid metabolism, and skin development including epidermis, hair follicle, and blood vessel development (Figure 1f,h). For example, proteins associated with skin barrier function (C8G, LGALS3, CASP14, and FLG2), epidermal development (TP63, KRT79, DSC3, DSG3, etc.), hair follicle development (KRT73, KRT28, KRT17, KRT80, KRT15, etc.), sweat gland development (KRT77 and LTPR3), basement membrane assembly (SAP18, LAMB3, LAMC2, LAD1, etc.), blood vessel development (RBM10, PDCD10, GTF2I, SERPINB5, etc.), amyotrophy (FRG1, UBQLN4, POLR1C, PNKP, etc.), oxidation‐reduction (LHPP, GALE, COX6A1, BBOX1, etc.), lipid metabolism (PRKAR2B, ACSBG1, CRABP2, HACL1, etc.), and key signal pathways (Wnt signal and NF*κ*B signal) of skin development were downregulated in scleroderma mouse skin (Figure 1h). These proteomics characteristics were consistent with the pathological findings mentioned above. Besides, several regulatory ECM proteins were downregulated, including lysosomal cysteine proteinases (CTSA, CTSB, CTSC, CTSD, CTSH, CTSK, and CTSL), proteinase inhibitors (CSTB, SERPINB12, SERPINB5, and SERPINB8), and secreted factors (FLG2, IL1RN, S100A1, S100A11, S100A14, and S100A16) in scleroderma mouse skin (Figure S3b and Table S3, Supporting Information). These results indicate that dysregulation of regulatory factors in the microenvironment is indispensable for the pathological changes of skin in mice with scleroderma.

### 2.2 Establishment of iPSC‐derived Epidermal and Mesenchymal‐like (EM) Organoid Model

**Figure 2:**
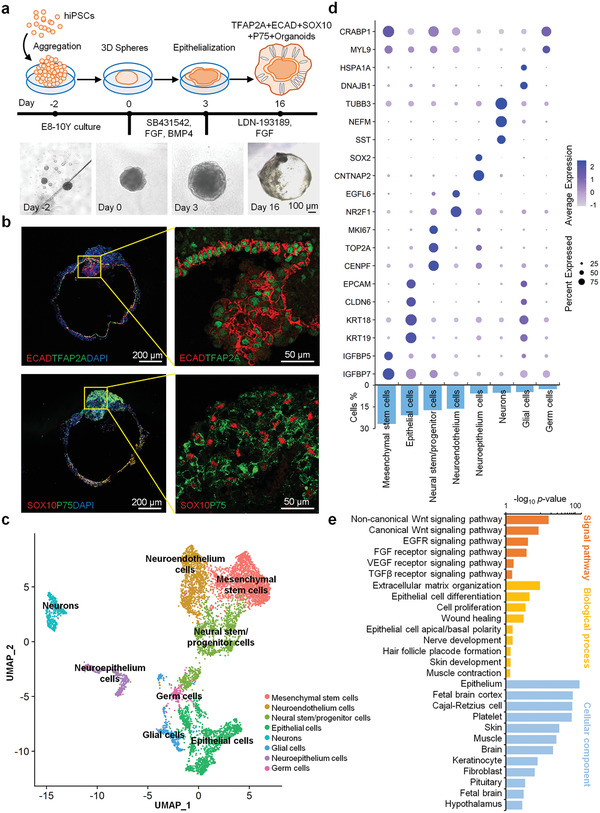
Formation of skin organoids in vitro. a) Schematics of the induced pluripotent stem cell (iPSC)‐derived organoid. (scale bar: 100 µm). b) ECAD, TFAP2A, SOX10, and P75 expression analysis of iPSC‐derived organoids (scale bar: 100 and 50 µm). c) Uniform manifold approximation projection (UMAP) plots of scRNA‐seq data of day 16 EM organoids. A total of 7392 cells were represented. The major cell groups were manually annotated and labeled with different colors. d) Expressions of the key gene markers and percentage distribution of different cell clusters. The statistical significance of cell type marker genes was performed using the Wilcoxon test with BH adjusted *p*‐value < 0.01. e) Signal pathway, biological process, and cellular component analysis of the day 16 organoids proteomic profile.

Following a previous study,^[^ ^11^ ^]^ we applied iPSCs to generate EM organoids. After aggregating iPSCs on U‐bottom 96‐well plates, we cultured the aggregates in Matrigel supplemented with bone morphogenetic protein 4 (BMP4), SB431542 (a TGF*β* inhibitor), and basic fibroblast growth factors (FGF). After 3 d of differentiation, the organoids were treated with LDN‐193189 (a BMP inhibitor) and FGF and incubated on a shaker (**Figure** **2**a). After 16 d, the organoids expressed epidermal markers (TFAP2A+ECAD+, Figure 2b). In addition, two subtypes of cranial neural crest cells appeared with mesenchymal (PDGF*α*+, Figure S4, Supporting Information) and neuroglial markers (SOX10+P75+, Figure 2b). To gain insight into the cell lineages that arise in this EM organoid model, we performed single‐cell RNA sequencing (scRNA‐seq) on day 16 organoids. The scRNA‐seq data clustering and gene expression analysis revealed that the day 16 organoids were mainly annotated into eight cell types, including MSCs (26.73%), epithelial cells (20.85%), neural stem/progenitor cells (17.15%), neuroendothelium cells (16.48%), neuroepithelium cells (5.75%), neurons (5.48%), glial cells (4.91%), and germ cells (2.65%) (Figure 2c,d and Figure S5a,b, Supporting Information). The epithelial cells (KRT19+KRT18+CLDN6+EPCAM+), and nerve cells were the other two main cell types of the EM organoids, in addition to the largest cell population MSCs (IGFBP7+IGFBP5+). Mainly, the nerve cells consist of neural stem/progenitor cells (CENPF+TOP2A+MKI67+), neuroendothelium cells (NR2F1+EGFL6+), neuroepithelium cells (CNTNAP2+SOX2+), neurons (TUBB3+NEFM+SST+), and glial cells (DNAJB1+HSPA1A+) (Figure 2d and Figure S5a,b, Supporting Information). The proteomics profile of the cellular components of the day 16 organoids was mainly enriched in the tissues of epithelium and brain—consistent with the above results (Figure 2e). Proteins of the organoids were mainly involved in the biological processes of ECM organization, epithelial cell, and nerve development (Figure 2e). Additionally, 212 ECM componets were identified in the EM organoids by scRNA data (Table S4, Supporting Information). Among them, proteins involved in tissue development (follistatin like 1 [FSTL1] and annexin A2 [ANXA2]), ECM remodeling (matrix metalloproteinase [MMP2]), serpin family E and F member 1 [SERPINE1 and SERPINF1], angiogenesis (secreted protein acidic and rich in cysteine [SPARC], lumican [LUM], decrin [DCN], thrombospondin 1 [THBS1]), collagen [COL8A2], and fibronectin [FN1]) were proved to promote the tissue repair of lung fibrosis.^[^ ^12^ ^]^ Besides, several ECM componets were found be associated with differentiation (semaphorin family members [SEMA3A, SEMA3D, SEMA3E, SEMA6A, SEMA6B, and SEMA6D]) and neurogenesis (neural EGFL like 2 [NELL2], netrin G1 [NTNG1], slit guidance ligand 2 [SLIT2], and neuron derived neurotrophic factor [NDNF]). Moreover, 130 ECM‐associated proteins, including 44 ECM regulators, 30 ECM‐affiliated proteins, and 56 secreted factors, were identified in the EM organoids. Among them, many secreted proteins including placental growth factor (PGF), vascular endothelial growth factor (VEGFA), growth factors (platelet derived growth factors [PDGFA and PDGFD], bone morphogenetic proteins [BMP4, BMP5, and BMP7], fibroblast growth factors [FGF17 and FGF18], and insulin like growth factor [IGF1 and IGF2]) were identified in the organoids. Skin development associated noncanonical and canonical Wnt signal pathways including Wnt family members [WNT4, WNT6, WNT7B, WNT8B, and WNT10A], Wnt interacted proteins [SFRP1, SFRP2, and FRZB], WNT inhibitory factor [WIF1], Wnt signaling pathway activators [RSPO1, RSPO2, and RSPO3], as well as growth receptor and factors (EGFR, FGF, VEGF, and TGF*β*) were also found be enriched in EM organoids (Figure 2e and Figure S5c, Supporting information). These findings indicate that we have successfully constructed EM organoids composed of MSCs, epithelium, and nerve‐like cell types with diverse cellular functions.

### 2.3 EM Organoids Rescued Skin Fibrosis and Inflammation in Scleroderma Mice

**Figure 3:**
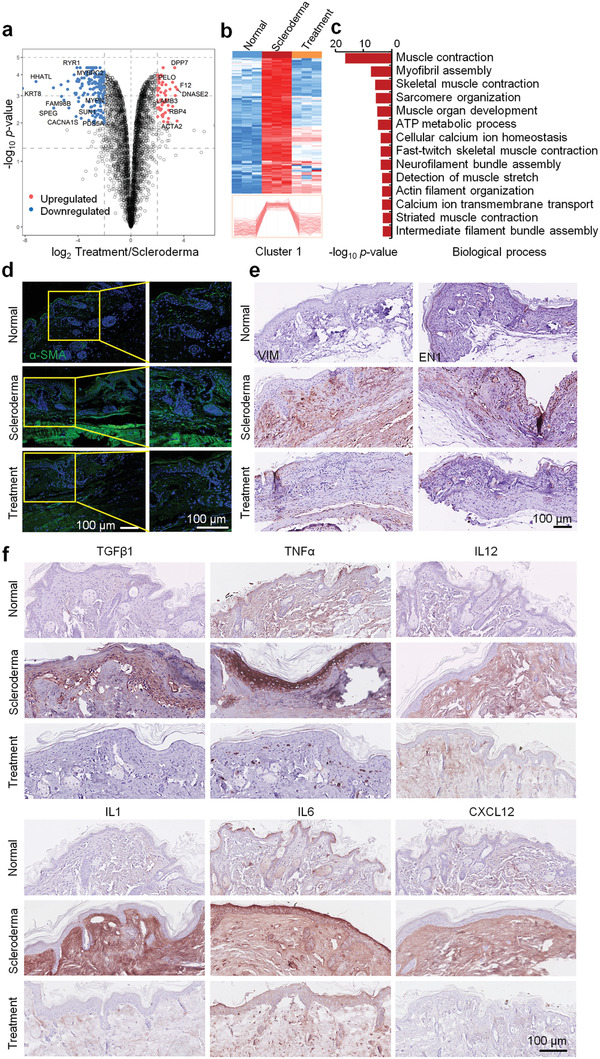
EM organoids rescued skin fibrosis and inflammation in scleroderma mice. a) Volcano plot of the log_10_ *p*‐value versus the log_2_ protein abundance comparison between the scleroderma and organoid treatment groups. Proteins outside the significance threshold lines (−log_10_ *p‐*value > 2, log_2_ Treatment/Scleroderma > 1 or < −1) are shown in red (upregulated) and blue (downregulated). b) Heatmap represents the differentially expressed proteins in skin from the normal (*n* = 3), scleroderma (*n* = 3), and organoid treatment (*n* = 3) groups. Protein expressions in Cluster 1 were upregulated in scleroderma (compared to the normal group) and recovered by organoid treatment. c) Biological process analysis of enriched proteins in Cluster 1. d) Immunofluorescence and e,f) immunohistochemistry analysis of *α*‐SMA, VIM, EN1, TGF*β*1, TNF*α*, IL12, IL1, IL6, and CXCL12 in normal, scleroderma, and organoid treatment skin tissues (scale bar: 100 µm).

The scleroderma mice were transplanted with EM organoids for one month (Figure 1a). The proteomics analysis on the treated skins showed that all proteins could be classified into three groups based on the proteomics profiles generated using the principal coordinate analysis (Figure S6a,b, Supporting Information). In total, 614 proteins were differentially expressed between the treatment and scleroderma groups (Figure 3a and Table S5, Supporting Information). Further analysis focused on the differentially expressed proteins in the scleroderma, exhibiting restored expression after organoid treatment. Cluster 1 represented upregulated proteins in the scleroderma group and got downregulated after treatment (Figure 3b). These proteins were highly enriched in the biological processes of muscle contraction and development, myofibril assembly, and ATP metabolism, indicating that muscle fibrosis was effectively ameliorated with the EM organoid treatment (Figure 3c). Pathological stainings also showed that the expressions of fibrotic markers (*α*‐SMA, VIM, and EN1) were significantly downregulated in the skin tissues of scleroderma mice after EM organoid treatment (Figure 3d,e). In addition, we evaluated the relevant infoammatory cytokines of IL1, IL6, IL12,TNF*α*, and CXCL12 in the normal, scleroderma and treatment groups, respectively. We found high levels of these cytokines in the scleroderma group, compared with the normal group (Figure 3f). Then, the expression levels of these cytokines downregulated in the treatment group, compared to the scleroderma group. These results indicate that EM organoids can rescue skin fibrosis in scleroderma mice.

### 2.4 EM Organoids Promoted the Tissue Repair in Scleroderma Mice

**Figure 4:**
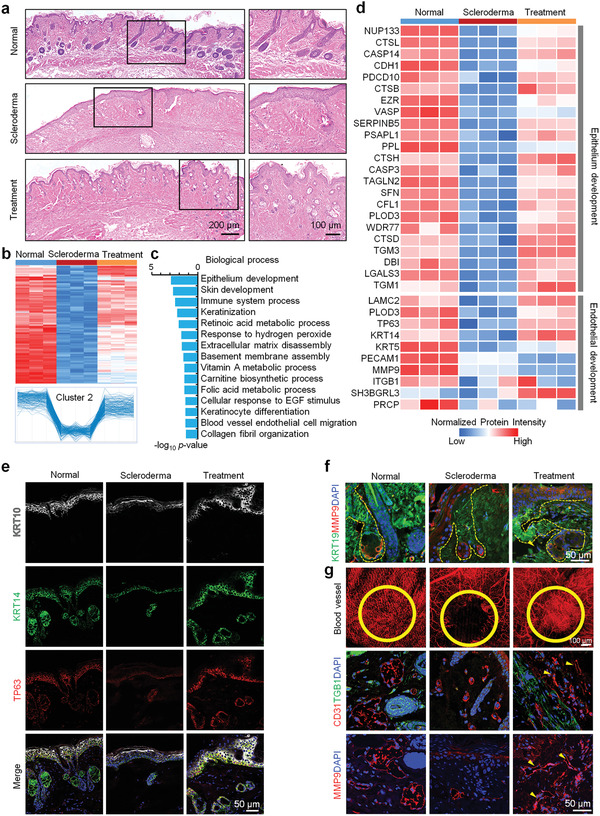
EM organoids promote the regeneration of epidermis, epidermal appendages, and blood vessels in scleroderma mice. a) H&E stainings of skin tissues from the normal, scleroderma, and organoid treatment groups (scale bar: 200 and 100 µm). b) Heatmap representing the differentially expressed proteins in skin from the normal (*n* = 3), scleroderma (*n* = 3), and organoid treatment (*n* = 3) groups. Protein expressions in Cluster 2 were downregulated in scleroderma (compared to the normal group) and were recovered by organoid treatment. c) Biological process analysis of enriched proteins in Cluster 2. d) Heatmap represents the expression levels of proteins associated with epithelium and endothelial development in the normal (*n* = 3), scleroderma (*n* = 3), and organoid treatment (*n* = 3) groups. Gene names are presented on the left side of the heatmap. Red and blue boxes indicate the normalized intensity of enriched and depleted proteins, respectively. Immunofluorescence stainings of e) KRT10, KRT14, and TP63 in skin tissues and f) KRT19 and MMP9 in sweat glands from the normal, scleroderma, and organoid treatment groups (scale bar: 50 µm). g) Blood vessel analysis and CD31, TGFB1, and MMP9 expressions in skin tissues from the normal, scleroderma, and organoid treatment groups (scale bar: 50 and 100 µm).

Usually, the overproduction and accumulation of collagens appear in the LoS tissues. To test the therapeutic effect of EM organoid treatment on the LoS, hematoxylin and eosin (H&E) and Masson staining were performed to quantify the dermal thickness of the normal, scleroderma, and treatment groups. We observed that the dermis of scleroderma mice became thicker and had more collagens than the normal groups (Figure S6c,d, Supporting Information). However, in the treatment group, the thickness of the LoS skin tissues returned to a near‐normal level. Further, we evaluated the type I, II, III, VI, and XII collagens associated with fibrosis of LoS in normal, scleroderma, and treatment groups, respectively. High expression levels of these collagens were presented in the scleroderma group, compared with the normal group (Figure S6e, Supporting Information). Excitedly, the expression levels of these collagens were downregulated in the treatment group, compared to the scleroderma group (Figure S6e, Supporting Information). The previous results showed that the signal pathways and key proteins associated with epidermal development in scleroderma mice were seriously downregulated.^[^ ^13^ ^]^ Furthermore, pathology findings revealed that sweat glands and hair follicles were almost non‐existent in scleroderma group (Figure 4a), which was consistent with a previous report.^[^ ^14^ ^]^ The cluster 2 proteins that represented downregulated proteins in the scleroderma group and became upregulated after EM organoid treatment (Figure 4b). These proteins were highly enriched in epithelium development and keratinization processes, as well as ECM disassembly and basement membrane assembly (Figure 4c,d). Immunofluorescence stainings revealed that the expression levels of epidermal stem cell markers (KRT14 and TP63) and mature epidermal marker (KRT10) were downregulated in scleroderma, while these proteins were recovered to normal levels in the treatment group (Figure 4e). Additionally, sweat glands were found regenerated in the skin of scleroderma mice after EM organoid treatment (Figure 4a). The atrophic KRT19+ sweat glands in scleroderma mice recovered to a normal level in the organoid treatment group than in the untreated group (Figure 4f and Figure S6f, Supporting Information). These results indicate that the epidermis and its appendage‐associated functions can be recovered in scleroderma mice by EM organoid treatment.

Our results also showed that blood vessels of scleroderma group were injured, and especially the superficial vessels were almost absent in skin tissues of scleroderma mice (Figure S1d, Supporting Information). Functional analysis showed that the proteins associated with the blood vessel development were downregulated in scleroderma mice (Figure 1h). In contrast to the untreated scleroderma mice group, the ability of endothelial development associated proteins (LAMC2, PLOD3, and TP63) recovered in the treatment group (Figure 4d). Also, the blood vessels were regenerated in the skin tissues of EM organoid treated mice (Figure 4g). The expressions of endothelial cell adhesion markers (platelet and endothelial cell adhesion molecule 1 [CD31] and integrin subunit beta 1 [ITGB1]) and angiogenesis‐associated matrix metalloproteinase nine (MMP9) were upregulated in the treatment group (Figure 4g), indicating that the EM organoids can promote skin angiogenesis in scleroderma mice.

## 3 Discussion

The clinical manifestation of scleroderma is heterogeneous in different disease development stages, including skin fibrosis, Raynaud's phenomenon, vascular disturbances, joint pain, digital ulcers, and telangiectasia. LoS usually happens in children and juveniles, with a mean age of 6.4–8.7 years.^[^ ^15^ ^]^ A higher frequency of deep tissue and extracutaneous involvement was found in the pediatric scleroderma, accompanied by arthropathy, uveitis, facial hemiatrophy, seizures, and neuropathy.^[^ ^16^, ^17^ ^]^ Although several strategies were adequate for most patients, 30% of juvenile LoS patients did not respond to the immunosuppressive therapy. There is a need for additional treatment strategies for patients with recurrence following treatment.^[^ ^18^, ^19^, ^20^ ^]^ In this study, we first established a mouse model of LoS to investigate the pathological characteristics of LoS. Results showed that the phenotypes of skin lesions in scleroderma mice were diverse, including inflammatory cell infiltration, vascular atrophy, and severe fibrosis. Moreover, we found that the epidermal development of scleroderma skin tissue was downregulated, especially the epidermal appendages, such as sweat glands and hair follicles, almost disappeared. Proteins associated with the function of the epidermis and its appendages were significantly downregulated, indicating the need to focus on the dysregulation of epidermal function and dermal fibrosis, besides the immunotherapy in the treatment of localized scleroderma.

Next, we established a hiPSC‐derived EM organoids for LoS treatment. For a long time, MSCs have been used as an ideal stem cell therapy to restore immune function due to their functional characteristics such as pro‐angiogenic, anti‐inflammatory, and immunomodulatory property. The scRNA‐seq results revealed that EM organoids contained 26.73% of the MSCs population. Results that the inflammatory cells in the scleroderma mice skin decreased significantly after EM organoids treatment indicated that the EM organoids can regulate inflammation in LoS. Further, we found several inflammatory factors (IL1, IL6, IL12, TNF*α*, and CXCL12), which were overexpressed in the LoS and became downregulated in the EM organoids treated skin. Besides, EM organoids contained 20.85% epithelial cell population, which is very important for the functional recovery of the epidermis and its appendages in LoS skin tissues. Functional analysis showed that EM organoids contain various epidermal cell proliferation and differentiation proteins. Most of these proteins were expressed in skin tissues and participate in important signal pathways to develop the epidermis and its appendages, such as Wnt, EGFR, and FGF signal pathways. After EM organoids treatment, we found the activity of epidermal stem cells was enhanced in the skin of LoS mice, and the sweat glands and hair follicles increased significantly. In addition, the ability of angiogenesis in LoS skin tissues was significantly enhanced, and it could be attributed to nerve endothelial cells (16.48%) in the EM organoids cell population. Besides, we also identified those vascular endothelial cell migration‐associated proteins and several key pathways of vascular regeneration in the EM organoids. More than, various ECM proteins were identified including some cytokines and regulated factors that could promote tissue repair in the EM organoids. These results suggest that the potential mechanism of skin tissue repair in LoS mice is to promote injury repair through active factors secreted by organoids.

Central nervous system (CNS) involvement was also found in juvenile LoS, with neurological manifestations, possibly due to autoimmune affection.^[^ ^21^, ^22^ ^]^ It was speculated that the CNS disease of LoS was originated from neuroectoderm, and the face and brain tissues was derived from a common progenitor of the ectoderm.^[^ ^23^ ^]^ The EM organoids contained PDGFR*α*+ and SOX10+p75+ cranial neural crest cells, including 17.15% neural stem/progenitor cells, 5.48% neurons, and 4.91% glial cells, according to the scRNA‐seq results. Proteins associated with nerve development were also enriched in the EM organoids, indicating that EM organoids have the potential to promote the repair of the CNS in LoS.

Another problem to address is how to determine the best treatment window for organoids therapy on LoS and obtain the beneficial effects. We chose the mouse model of scleroderma to treat bleomycin for one month because scleroderma can be controlled as localized scleroderma during this period. In addition, organoid therapy in this period can effectively alleviate fibrosis and inflammation of scleroderma. Nevertheless, determining the best treatment window will be an important experimental project that must be carried out in preclinical experiments in the future. In addition, one big challenge in translating iPSC therapy is safety and teratoma formation. In our experiment, we examined the mice with transplanted organoids after three months and did not observe any tumorigenesis, concluding that there were no human‐derived cells in the skin tissues, indicating the safety of EM organoid transplantation.

In conclusion, this study provides explicit evidence that the application of iPSC‐derived EM organoids can promote recovery of scleroderma pathological phenotypes and skin‐associated function, revealing a new feasible method for treating scleroderma from the perspective of regenerative medicine.

## 4 Experimental Section

### hiPSC‐Derived Organoid Culture

The hiPSC line (Human iPSC Line nciPS02, RC01001‐B, Female, Nuwacell Biotechnologies Co., Ltd.) was cultured on six‐well plates coated with 1% Matrigel (BD). Pluripotent stem cells were maintained in mTeSR1 (Stem Cell) medium supplemented with 100 µg mL^−1^ Normocin (Invivogen) coated with 1% Matrigel (Corning). For differentiation, hiPSC colonies were detached from the culture dish using Accutase Cell Dissociation Reagent (Gibco). Then, cells were collected as a single‐cell suspension in Essential 8 Flex (Gibco) medium containing 20 × 10^−6^ m Y27632 (Selleck). Cells were distributed at a density of 3500 cells in 100 µL of medium per well into 96‐well U‐bottom plates (Corning). After 24 h of incubation at 37 °C, fresh E8 medium without Y27632 was added to a total volume of 200 µL per well, and the plates were incubated at 37 °C continuously. Next, cell aggregates were cultured in E6‐based differentiation medium containing 2% Matrigel, 10 × 10^−6^ m SB431542 (Selleck), 4 ng mL^−1^ basic FGF (hereafter FGF; R&D Systems), and 2.5 ng mL^−1^ BMP4 (R&D Systems). On day 3 of differentiation, 200 ng mL^−1^ LDN‐193189 (BMP inhibitor, Selleck) and 50 µg mL^−1^ FGF were added to induce cranial neural crest cell formation. On day 12, all cell aggregates were transferred to 24‐well low‐attachment plates (Thermo Fisher Scientific) in 500 µL of organoid maturation medium (composed of nutrient mixture F‐12 [DMEM/F‐12; Gibco] and neurobasal medium [Gibco] at a 1:1 ratio, 1× GlutaMax [Gibco], 0.5× B‐27 minus vitamin A [Gibco] and 0.5× N‐2 [Gibco] supplements, 0.1 × 10^−3^ m 2‐mercaptoethanol [Gibco], and 100 µg mL^−1^ Normocin) containing 1% Matrigel to induce self‐assembly of the epidermis. Half of the medium was replaced after 16 d after differentiation. On day 17, the organoids were collected for the subsequent experiments.

### Animal Models and Organoid Transplantation

All experimental work about the animals was approved by the Institutional Animal Care and Use Committee of the Military Academy of Medical Sciences, Beijing, and performed in accordance with its guidelines (approval No. IACUC‐DWZX‐2020‐689). Bleomycin (BLM) powder was dissolved in PBS buffer at a concentration of 2 mg mL^−1^ and sterilized via filtration. To establish the scleroderma animal model, nude mice (6 weeks old, weighing 22–24 g, male) were subcutaneously injected daily with 50 µL of BLM solution (2 mg mL^−1^) at a single location on their shaved backs (1 cm^2^) for four weeks using a 27‐gauge needle. As a control experiment, physiological saline without BLM was injected into age‐matched nude mice. All microvascular images were acquired by the Monitoring System of Vascular Microcirculation in Vivo (Micro‐VCC, OPTOPROBE, BeiJing HealthOLight Technology Co., Ltd.).

The pathology of BLM induced localized scleroderma was examined via histological analysis in the scleroderma skin tissues. Anesthetized mice were placed on a preheated electric blanket. Then, a 3–5‐cm incision was made into the back of each mouse. Each incision was transplanted with 3–5 organs grown for 16 d, followed by four weeks of acclimatization. The wound was sutured with crochet and fine thread, and vital signs were assessed immediately and 2, 5, and 8 h after transplantation. All mice received continuous BLM injections. Four weeks later, the skin of the injection site of each mouse was collected for blood vessel imaging using the Monitoring System of Vascular Microcirculation in Vivo (Micro‐VCC, Optoprobe Science LTD, UK). Mouse skin tissues were collected for pathological staining and proteomics analysis.

### Pathological Staining Analysis

Skin tissues were fixed in 4% formaldehyde overnight at 4 °C and then processed using a dehydration gradient. After tissues were embedded in paraffin, the sections were cut at a thickness of 4 µm for H&E staining. For immunofluorescence staining, sections were fixed in 4% formaldehyde for 20 min and washed in PBS. Then, sections were treated with 0.25% Triton X‐100 for 20 min at 26 ℃. Then, sections were incubated with primary antibodies overnight at 4 °C. After incubation for 1 h at 26 °C with secondary antibodies and counterstaining with DAPI, sections were sealed with Fluoro‐Gel for Photography. Negative control samples were incubated with secondary antibody alone. The pictures were taken at 20×/40× magnification using a confocal microscope.

### Confocal Microscopy

Immunofluorescence imaging was used to observe epithelial and mesenchymal characteristics, biomarkers of blood vessels and sweat glands, and the characteristics of fibrosis. Materials were fixed in 4% formaldehyde for 20 min and washed in PBS. Samples were then treated with 0.5% Triton X‐100 for 15 min, subsequently blocked with 10% serum for 1 h at 26 °C, and incubated with primary antibodies overnight at 4 °C. After incubation for 1 h at 26 °C with secondary antibodies and counterstaining with DAPI, images were taken using an LSM 700‐point scanning confocal microscope equipped with 5× and 10× objectives and adjusted linearly for presentation.

### Masson's Trichrome Stain

The paraffin sections were first dewaxed with concentrations of ethanol solutions, stained with Weigert's iron hematoxylin solution for 5–10 min, and differentiated with acid alcohol differentiation solution for 10–15 s. Blue in Bluing solution for 2–5 min, rinsed in deionized water and subsequently stained with Ponceau‐acid fuchsin solution for 5–10 min, rinsed in deionized water, differentiated in phosphomolybdic acid solution for 1–2 min or until collagen was not red. Then, without rinsing, aniline blue solution was added to the sections for 1–2 min, which were that placed in acetic acid working solution for 1 min. Finally, the sections were very quickly dehydrated in 95% ethanol, absolute ethanol, transparent in xylene, and sealed with resins.

### Mass Spectrometry

Mouse skin tissue samples were homogenized at 4 ℃. After centrifugation at 14 000× *g* for 10 min at 4 °C, the supernatants were reduced by adding tributylphosphine (final concentration, 5 × 10^−3^ m), followed by vortexing for 10 min at room temperature. After centrifugation at 14 000× *g* for 30 min at room temperature, supernatants were transferred to a clean tube, and 100‐µg samples were added into 10 kDa ultrafiltration tubes containing 400 µL of urea buffer (8 m urea, 150 × 10^−3^ m Tris HCl, pH 8.0). After centrifugation at 12 000 rpm for 10 min at room temperature, the liquid in the collection tube was discarded, and the aforementioned steps were repeated three times. Next, 25 µg of the collected samples were solubilized at 37 °C for 4 h in 1 m dithiothreitol. Iodoacetamide (1 m) was added to the samples, which were incubated in the dark for 30 min at 25 ℃. The samples were centrifuged at 12 000× *g* for 5 min at room temperature, and the supernatants were removed. Next, 100 µL of uric acid were added, samples were centrifuged twice at 12 000× *g* for 5 min each at room temperature, and the supernatants were removed. NH_4_HCO_3_ (50 × 10^−3^ m) was added to the samples, and the supernatants were removed after centrifugation at 12 000× *g* for 5 min at room temperature. Final digestion was performed at 37 °C overnight via incubation with trypsin (1:50 enzyme/substrate). After centrifugation at 14 000× *g* for 30 min, the supernatants were transferred to clean tubes for LC‐MS/MS analysis. The peptide mixtures were analyzed using an Orbitrap Fusion Tribrid Mass Spectrometer equipped with an Easy‐nLC nanoflow liquid chromatography system.

### Proteomics Data Processing

The mass spectrometry raw files were searched against the SwissProt mouse sequences (downloaded on February 8, 2021, containing 17 056 proteins) and common contaminants using MaxQuant software (version 1.6.5.0).^[^ ^24^ ^]^ Peptides were identified using a precursor mass tolerance of 4.5 ppm and a fragment mass tolerance of 20 ppm. Cysteine carbamidomethylation was set as the fixed modification, and N‐terminal acetylation and methionine oxidation served as variable modifications. Up to two missed cleavages were allowed, and trypsin was set as the enzyme specificity. Automatic target and reverse database searches were used, and a false discovery rate of 1% was allowed at both the peptide and protein levels. Protein quantification was performed using the iBAQ quantification method^[^ ^25^ ^]^ in MaxQuant.

### Skin Organoid Dissociation and scRNA‐seq Sequencing

The day‐16 EM organoids (*n* = 20) were collected for generating single‐cell gene‐expression libraries. The collected organoids were incubated with TrypLE for 10–20 min at 37 °C with gentle swirl, until the organoids dissociated into a single‐cell suspension with no obvious cell aggregation. After neutralizing trypsin with bovine serum albumin (BSA) solution, the suspension was filtered through a 40‐µm Flowmi cell strainer to remove cell debris. After three washes with cold 3% BSA solution, cells were resuspended in 3% BSA. Through Trypan blue staining, cell viability and live cell count was calculated to ensure that the final cell concentration and viability were applicable for sequencing. Then, the single‐cell 3′ RNA‐seq experiments were conducted using the Chromium single cell system (10× Genomics) and the Illumina NovaSeq 6000 sequencer (S4 V1.5).

### scRNA‐seq Data Analysis

The Cell Ranger software (10× Genomics) (https://support.10xgenomics.com/, version 6.1.1) was used to process the scRNA‐seq raw data. The FASTQ files are aligned to the human genome and transcriptome (hg38) to generate the gene expression matrix. The R package Seurat (version 4.0.2) was then applied for further data analysis. Cells with less than 1000 genes or more than 4000 detected genes were filtered, and those with more than 10% mitochondrial genes were further excluded. Then, 7392 cells were remained. After log‐normalization, top 2000 highly variable genes were determined using the “FindVariableFeatures” function for downstream bioinformatics analyses. The top‐20 principal components of PCA were used to perform UMAP clustering. The “FindAllMarkers” function was used to identify cluster markers with the follow setting: min percentage of cell expressed = 0.25, log fold change threshold = 0.25, and min percentage of gene expressed = 0.1.

### Statistical Analysis

Statistical analyses were performed using R package (version 4.0.3). Each experiment was performed at least three times (*n* ≥ 3). The intensity values of all proteins identified by proteomics were normalized by taking the fraction of the total, followed by multiplication of 10^6^ and log2 transformation. Pairwise comparisons were performed by a moderated t‐statistic using the R package Limma (version 3.38.3) to identify the differentially expressed proteins among the three experimental groups. Differentially expressed genes between two groups of cells in scRNA‐seq data were identified using a Wilcoxon Rank Sum test. Differences for which the Benjamini–Hochberg adjusted *p*‐value was less than 0.01 were considered statistically significant. Protein and gene identifications were annotated using the online tool DAVID (https://david.ncifcrf.gov/)^[^ ^26^ ^]^ according to the GO annotation of biological processes. Heatmaps of the protein quantitation values were constructed using the Perseus software (version 1.6.10.50).^[^ ^27^ ^]^ The protein–protein interactome network was built using Cytoscape (version 3.8.2),^[^ ^28^ ^]^ and the protein–protein associations were retrieved from the STRING database.^[^ ^29^ ^]^ The Matrisome database^[^ ^10^ ^]^ (http://matrisomeproject.mit.edu/) was used to annotate the ECM components. The circlize package^[^ ^30^ ^]^ (version 0.4.11) was used for circular visualization.

## Conflict of Interest

The authors declare no conflict of interest.

## Author Contributions

J.M., W.L., R.C., and D.G. contributed equally to this work. L.L., J.M., S.Z., and W.Z. conceived the overall study and designed experiments. L.L., S.Z., and W.Z. had full access to all the data in the study and take responsibility for the accuracy of the data analysis. L.L., D.G., Q.Z., L.Lv, and R.C. contributed to the culture of skin organoids. W.L., R.C., D.G., and B.L. constructed the scleroderma animal model. J.M., L.L., X.L., M.L., and Y.Z. performed proteomics and scRNA‐seq experiments and bioinformatics analysis. L.L., D.G., L.Lv, Y.W., J.J., and Z.W. performed most of pathological staining experiments. L.L., J.M., W.L., R.C., S.Z., and W.Z. wrote and edited the manuscript. W.Z., S.Z., Y.Z, and Z.W. provided funding support. All authors made important comments to the manuscript.

## Supporting information

Supporting Information

Supplemental Table 1

## Data Availability

The data that support the findings of this study are openly available in ProteomeXchange Consortium at http://proteomecentral.proteomexchange.org/, reference number PXD025613.
