# Supplementary material for: Application of an iPSC‐Derived Organoid Model for Localized Scleroderma Therapy
Source: Adv Sci (Weinh). 2022 Mar 22;9(16):2106075. doi: 10.1002/advs.202106075 (PMC9165518; doi:10.1002/advs.202106075)
Supplement: Supplementary file 1 — Supporting Information [file ADVS-9-2106075-s001.pdf]

Supporting Information

Application of an iPSC-derived organoid model for located scleroderma therapy

Jie Ma†, Wei Li†, Ruiyuan Cao†, Dunqin Gao†, Xiao Li, Luye Lv, Biyou Li, Xiaoqing Liu, Mansheng Li, Junyi Jiang, Qiyu Zhang, Yujie Wang, Zhihong Wu, Yunping Zhu, Wu Zhong*, Shuyang Zhang*, Ling Leng*

† These authors contributed equally.

* Correspondence to: lengling@pumch.cn (L.L.); shuyangzhang103@nrdrs.org (S.Z.); zhongwu@bmi.ac.cn (W.Z.).

Supplementary Figures

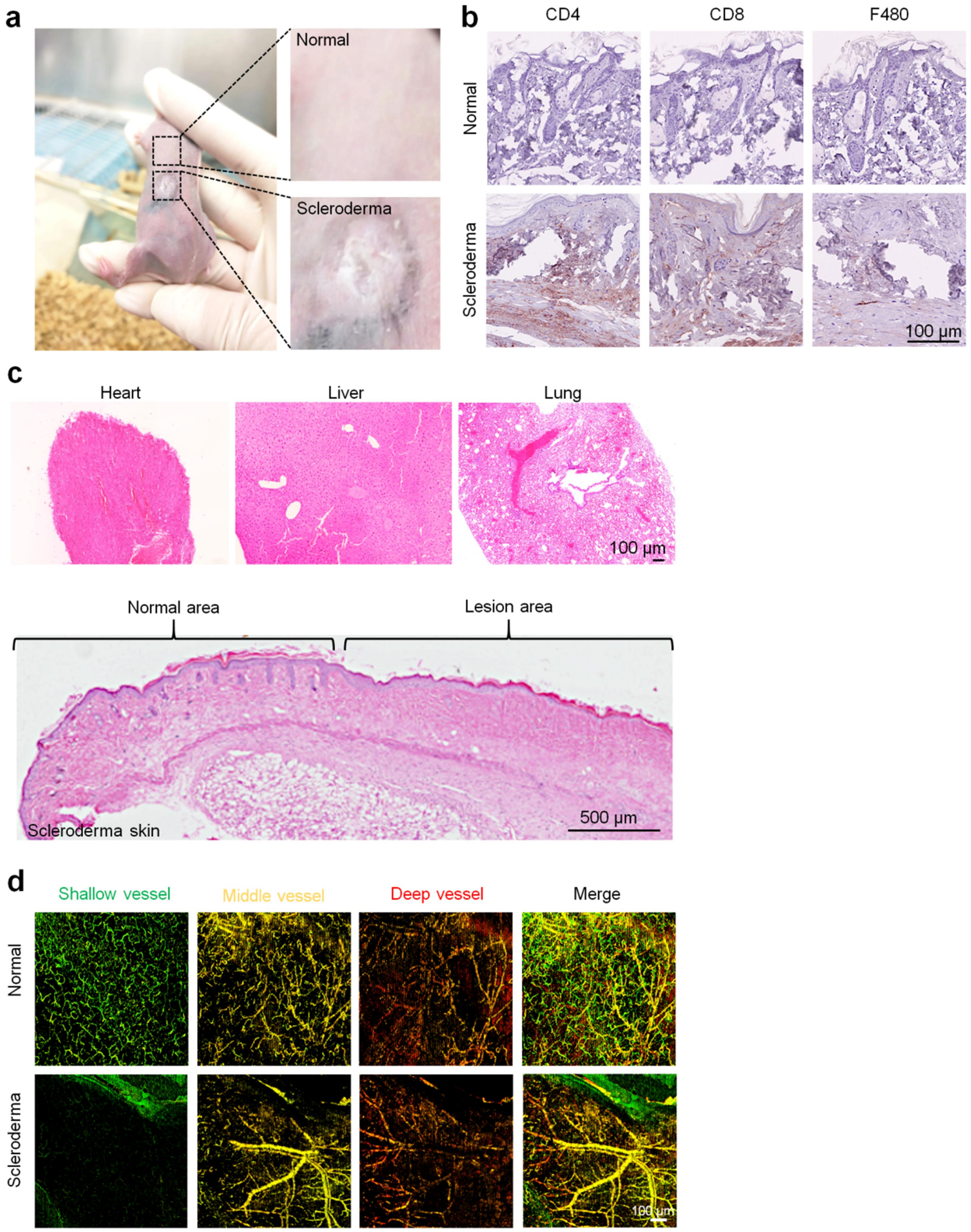

Figure S1. Pathological staining analysis of scleroderma mice. a) Photos of skin lesions of scleroderma mice. b) Immunohistochemical staining of CD4+T cells, CD8+T cells and F480+macrophages on scleroderma and normal groups (scale bar: 100 μm). c) Hematoxylin and eosin (H&E) staining of heart, liver, lung, and skin tissue from scleroderma mice (scale bar: 100 and 500 μm). d) In-vivo microvascular

imaging analysis for skin of scleroderma and normal mice (scale bar: 100 μm). Green, yellow, and red represent the blood vessels of the shallow, middle, and deep levels, respectively.

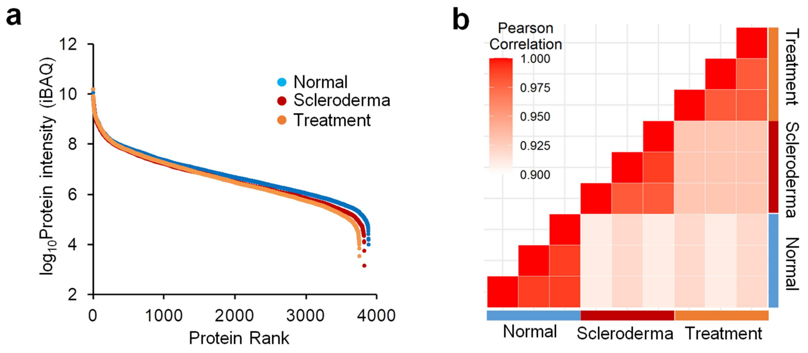

Figure S2. Proteomic profiles of scleroderma and normal mouse skin tissues. a) The protein intensity distributions of skin tissues in the normal, scleroderma, and organoid treatment groups. b) Correlation analysis of proteomic profiles of skin tissues in the normal, scleroderma, and organoid treatment groups.

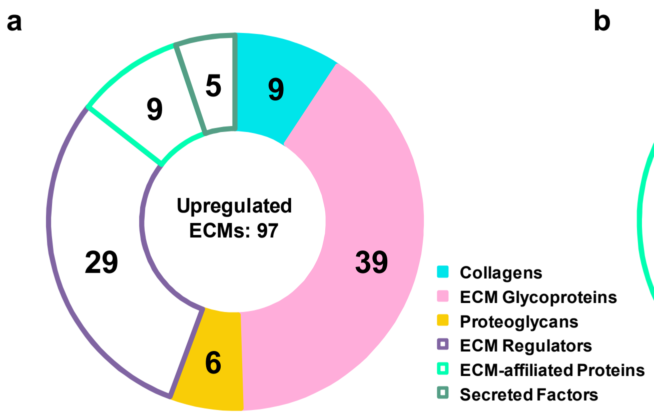

Figure S3. Matrisome profile analysis of extracellular matrix (ECM) proteins identified in the skin tissues of scleroderma mice and normal mice. Six types of ECM proteins (collagens, ECM glycoproteins, proteoglycans, ECM regulators, ECM-affiliated proteins, and secreted factors) that upregulated (a) and downregulated (b) in the skin of scleroderma mice versus normal mice were annotated according to the Matrisome database.

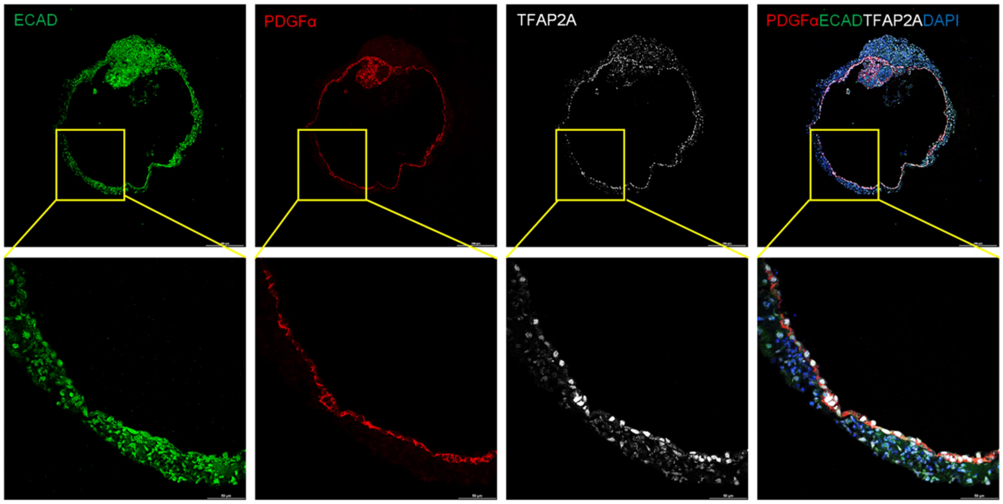

Figure S4. Formation of EM organoids. ECAD, PDGFα, and TFAP2A expression analysis of EM organoids (scale bar: 200 and 50 μm).

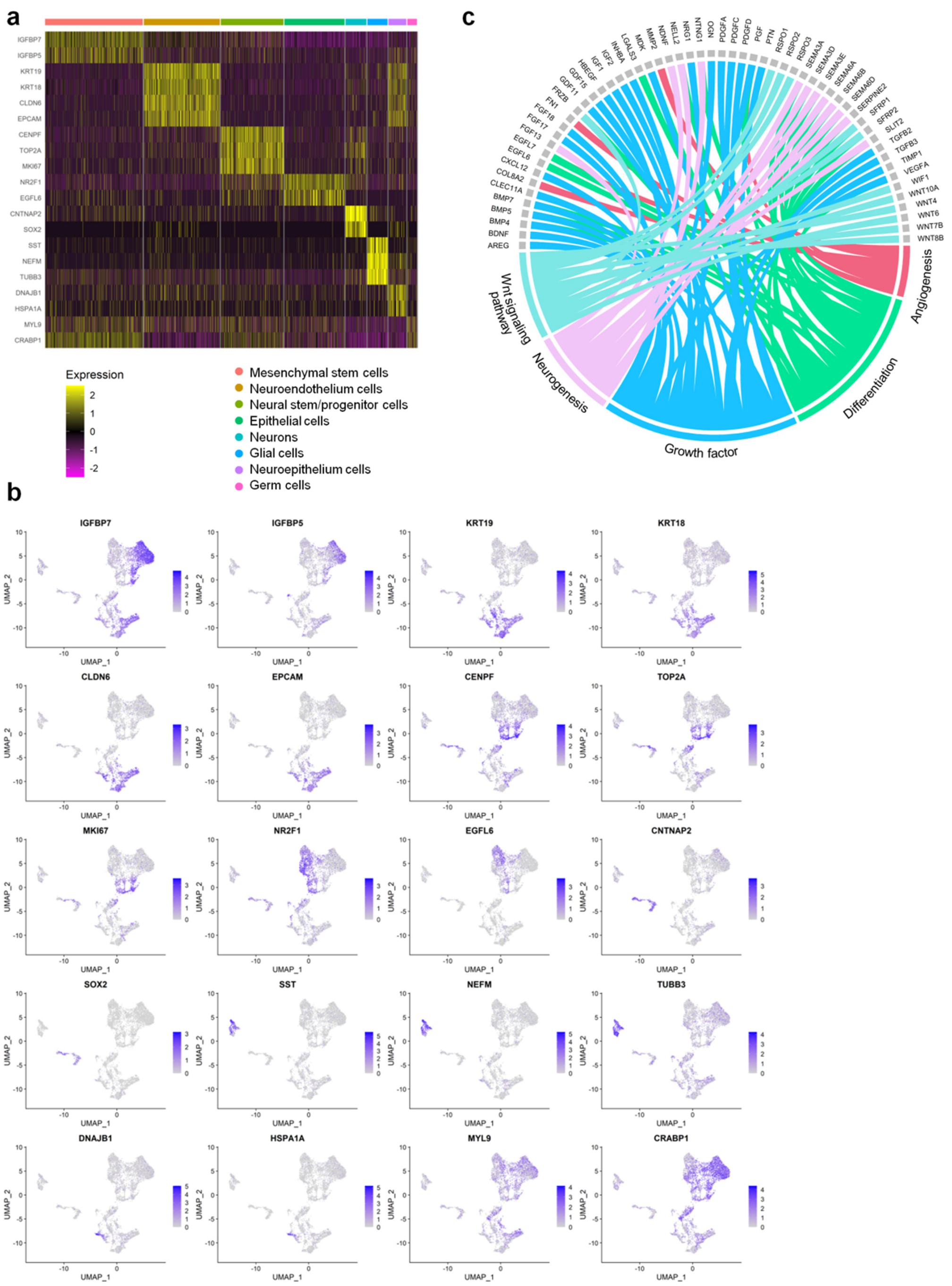

Figure S5. scRNA-seq gene expression signatures for different cell subtypes of day 16 EM organoids. a) Heatmap of the normalized expression of the top 10 differentially expressed genes per cell cluster for the day 16 EM organdies dataset. b) UMAP plots of the specific marker genes for the cell subtype classification. Differentially expressed genes between two groups of cells in scRNA-seq data are

identified using a Wilcoxon Rank Sum test. Differences for which the Benjamini–Hochberg (BH) adjusted *p*-value was less than 0.01 were considered statistically significant. c) Circos diagram of ECM components that are involved in multiple biological processes.

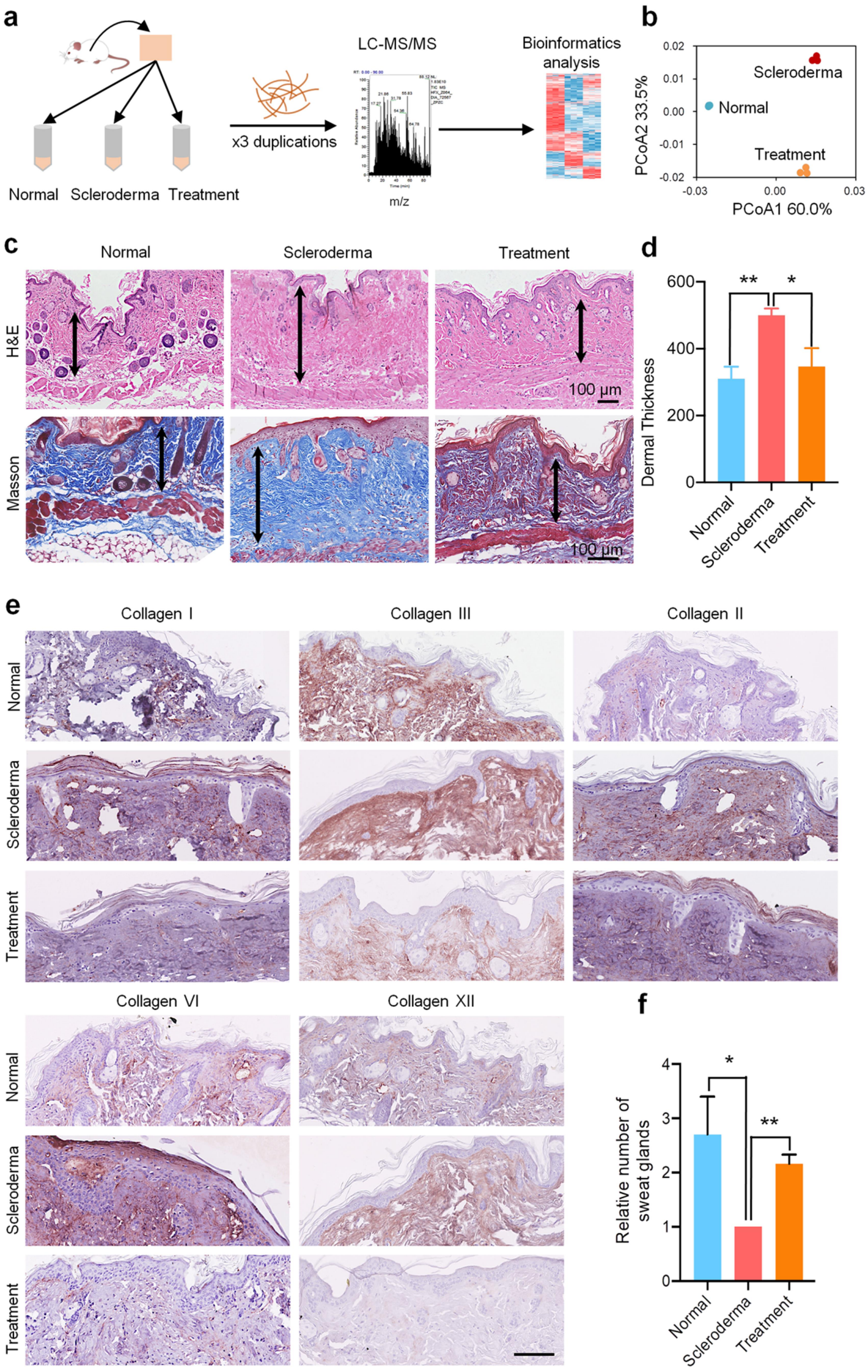
a
Normal
Scleroderma
Treatment
x3 duplications
LC-MS/MS
m/z
Bioinformatics analysis
b
PCoA2 33.5%
PCoA1 60.0%
Scleroderma
Normal
Treatment
c
H&E
Masson
100 µm
d
Dermal Thickness
e
Collagen I
Collagen III
Collagen II
Collagen VI
Collagen XII
f
Relative number of sweat glands

Figure S6. Proteomic profile of mice treated with organoids. a) Schematics of the proteomic analysis (n=3) used to evaluate skin tissues in the normal, scleroderma, and organoid treatment groups. b) Principal coordinate analysis (PCoA) of the proteome profile of all proteins within the three groups. Three technical repeats were produced for each skin sample, represented by different color points in the figure. c) Masson staing of skin tissues in the normal, scleroderma, and organoid treatment groups (Scar bar: 100 μm). The double arrows show the thickness of the leather. d) Quantitative analysis of dermal thickness in the normal, scleroderma, and organoid treatment groups. $n \geq 3$, t-test, * $p < 0.05$, ** $p < 0.01$. e) Immunohistochemical stainings of type I, II, III, VI, and XII collages in the normal, scleroderma, and organoid treatment groups (Scar bar: 100 μm). f) Quantitative analysis of the numbers of sweat glands in the normal, scleroderma, and organoid treatment groups. $n \geq 3$, t-test, * $p < 0.05$, ** $p < 0.01$.

Legends for Supplementary Table S1 to S6

Table S1. All Proteins identified in the skin tissues of normal, scleroderma, and organoid treatment mice.

Table S2. Differentially expressed proteins identified in the skin tissues of normal and scleroderma mice.

Table S3. All extracellular matrix components identified in the skin tissues of normal and scleroderma mice by proteomics.

Table S4. All extracellular matrix components identified in the 16 day human organoids by single cell transcriptome.

Table S5. Differentially expressed proteins identified in the skin tissues of scleroderma and organoid treatment mice.

Table S6. Detailed information about the reagents and equipments used in this study.
